# Effect of chemotherapy on autoimmune hepatitis in thymoma: a case report and literature review

**DOI:** 10.7497/j.issn.2095-3941.2013.03.008

**Published:** 2013-09

**Authors:** Nesrine Mejri, Imen Chabchoub, Ines Gargouri, Imtinen Belaid, Faten Ezairi, Sihem Hmissa, Slim Ben Ahmed

**Affiliations:** 1 Medical Oncology Department, Farhat Hached Hospital, Sousse 4011, Tunisia;; 2 Pathology Department, Farhat Hached Hospital, Sousse 4011, Tunisia

**Keywords:** Hepatitis, autoimmune, thymoma, chemotherapy

## Abstract

Autoimmune hepatitis (AIH) has rarely been described as an autoimmune paraneoplastic syndrome of thymoma. This case is the seventh case of AIH revealed by cholestasis few years after the diagnosis of thymoma and the first case treated with chemotherapy alone. We report in this paper a new approach to this rare severe condition. A 29 year-old man presented with chest pain and dyspnea with a history of thymoma surgically removed 4 years ago. CT scan showed the recurrence of an anterior mediastinal mass. Biology showed elevated liver enzymes and profound cholestasis. No sign of viral or toxic hepatitis or bile duct abnormalities were observed. Autoimmune antibodies, except for the anti-nuclear antibody, were negative. Liver biopsy showed active chronic AIH. The patient was diagnosed with recurrent thymoma with AIH and underwent 6 cycles of chemotherapy. A complete response on thymoma and cholestasis was obtained after 10 months of follow-up. Steroids and immunosuppressors are the standard treatment for AIH. The effect of chemotherapy as a specific treatment of this paraneoplastic syndrome needs to be considered.

## Introduction

Thymoma is the most frequent tumor of the thymus. The 2004 WHO classification considers three morphological types of thymoma according to genetic alterations (microsatellite instability, 6q25, 5q21-22 mutations): A, B (B1, B2, and B3), and AB. The B3 subtype is the most aggressive one with 50% overall survival within 5 years^1^. Median age at diagnosis is 50 years. Thymoma is a slow-growing tumor that can relapse after 10 years, implying lifelong follow-up. The rate of associated malignancy (e.g., lymphoma and lung sarcoma) is higher than that in the general population^2^. Many autoimmune paraneoplastic syndromes are associated with thymoma. The most popular such syndrome is myasthenia gravis, which occurs in 35% to 45% of cases. Other autoimmune diseases have been reported, such as systemic lupus erythematosus, Hashimoto’s thyroiditis, erythroblastopenia, type I diabetes mellitus, and in some cases, 2 or even 3 autoimmune diseases at the same time. Autoimmune hepatitis (AIH) associated with thymoma has rarely been reported, with fewer than 10 cases published in literature. AIH is a severe disease because it inhibits certain specific treatments of the primary tumor and destroys hepatic tissue and causes hepato-cellular failure.

We report a new case of AIH associated with thymoma. The new finding about this case is that chemotherapy reduces biological signs of hepatitis without need for steroids or immunosuppressors. Through this case report and a review of literature, we highlight the clinical and therapeutic aspects of this rare entity.

## Case report

**Figure 1:**
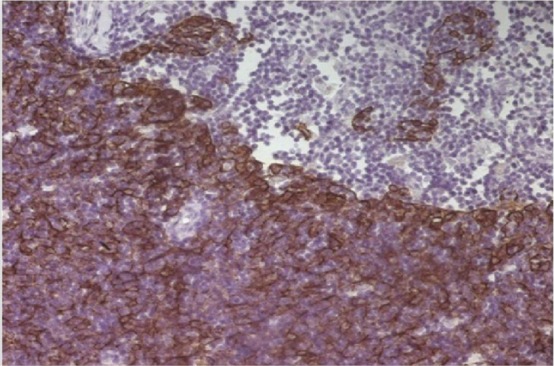
Type B thymoma, cytokeratine positive staining (IHC×40).

A 29 year-old male with dyspnea and chest pain was referred to our center. Medical history showed diabetes mellitus and B1 subtype thymoma (stage II) surgically removed 4 years ago (**Figure 1**). The patient did not receive adjuvant therapy at that time and was not followed up since.

Physical examination showed no signs of heart failure or myasthenia gravis. ECG was normal. Chest X-ray revealed an enlargement of the upper mediastinum with small pleural effusion. CT scan showed a tissular mass of the anterior mediastinum with dimensions of 3 cm × 5 cm × 6 cm. This mass reached the mediastinum medium and came into contact with the pericardium. Small pleural and pericardial effusion was observed. Cytology examination of pleural liquid was negative. Core biopsy was technically difficult and life-threatening. The patient exhibited local recurrence of a thymic tumor. Chemotherapy was decided, and the patient was admitted to our center in March 2012.

**Figure 2:**
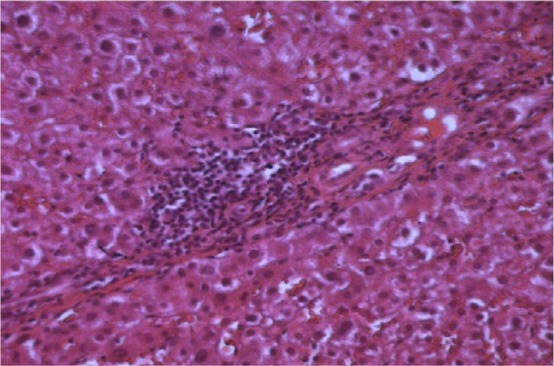
Lymphocyte infiltration and paracellular necrosis (H&E×40).

Blood cell count, renal function, and calcemia were normal. We observed a biological inflammatory syndrome with accelerated sedimentation speed (90 in the first hour) and polyclonal hyper gamma-globulinemia (46 g/L), predominantly of type IgG. Profound cholestasis without pruritis or jaundice was observed: total bilirubin at 72 mg/dL (5× normal), gamma glutamyl transferase at 1,680 UI/L (30× normal), and alkaline phosphatase at 780 UI/L (6× normal). The level of aspartate aminotransferase (ASAT) was 56 UI/L (1.5× normal), and that of alanine aminotransferase (ALAT) was 63 UI/L (1.5× normal). Blood sugar levels were disturbed. No history of drug abuse, including herb consumption, was reported. Abdominal ultrasonography showed no liver or bile duct abnormalities. Viral markers of hepatitis B and C were negative. Anti-nuclear antibody content was high (1/800, type ant-DNA), and anti-mitochondrial antibodies, anti-liver/kidney microsomes (LKM1), and anti-smooth muscles were negative. A liver biopsy showed signs of active periportal necrosis and fibrosis with an infiltration of inflammatory cells, mainly lymphocytes and plasmocytes (**Figure 2**). According to the scoring system of the International AIH Group, the pre-treatment score was 13, which corresponds to the probable diagnosis of AIH.

The patient was subjected to 3 cycles of chemotherapy based on cyclophosphamide, cisplatine, doxorubicine, and vincristine (CACV, every three weeks), with 50% reduction of vincristine doses because of hepatic cholestasis. Steroids were not indicated because the diabetes of the patient was difficult to stabilize and cytolysis was low and thus could not indicate immediate treatment. No grade III/IV toxicity was observed. Evaluation after 3 cycles showed clinical relief, radiological complete response on the thymic tumor, partial regression of pleural effusion, and 50% reduction of biological cholestasis (**Table 1**). The post-treatment score was 17, which corresponds to a definitive diagnosis of AIH. Diabetes was stabilized with insulin doses. The patient received 3 more cycles of the same chemotherapy. CT scan evaluation showed partial regression of the pleural effusion and normal liver lab values. The patient was considered to be in complete remission. He was recommended for mediastinal radiotherapy. At the time of this writing, the patient is asymptomatic with normal cholestasis markers and CT scan after 14 months of follow-up.

**Table 1:** Evolution of liver enzymes

Liver enzymes	Before chemotherapy	After 3 cycles of chemotherapy	After 6 cycles of chemotherapy
Gamma globulin (g/L)	46	35	24
Total bilirubin (mg/dL)	72	40	11
Gamma glutamyl transferase (UI/L)	1,680	1,050	230
Alkaline phosphatase (UI/L)	780	580	210
Aspartate aminotransferase (UI/L)	56	20	18
Alanine aminotransferase (UI/L)	63	22	20

## Discussion

Chemotherapy can be an efficient treatment of paraneoplastic disorders. This case is a combination of two autoimmune diseases associated with thymoma: type I diabetes mellitus, which occurred when thymoma was first diagnosed, and AIH. We cannot ensure that the diabetes was totally related to the thymoma, but based on chronological events and the absence of familial history, we considered it as a paraneoplastic syndrome. AIH was diagnosed with disease recurrence, but its onset was difficult to define. Signs of fibrosis suggest chronic evolution. All cases published in literature are summarized in **Table 2**. In these cases, AIH appeared a few months after the diagnosis of thymoma in 1 case^4^ but was synchronous to diagnosis in the other cases^3^^,^^5^. Most patients were from Asia, leading us to ask about genetic factors associated with high risk of AIH. Most patients were young, with a median age of 39 years. All 4 females and 2 males had no specific clinical presentation. AIH is generally revealed by biological liver dysfunction, with elevated liver enzymes (ASAT/ALAT). In our case, liver enzymes were slightly abundant, but major biological cholestasis was observed. The only case in literature on cholestasis associated with thymoma is related to autoimmune cholangitis^5^.

**Table 2:** Clinical and therapeutic aspects of cases reported in the literature

Reference	Age, yrs	Gender	Clinical presentation	Biology (UI/L)	Radiology	Pathology	Other paraneoplastic symptoms	Treatment	Evolution and follow up
Ko *et al.*^3^	25	F	Incidental	ASAT: 1158 ALAT: 943 PAL: 106 ANA+ SM– LKM1–	Not mentioned	Active chronic hepatitis	Myasthenia gravis Polymyosite	Prednisolone Aziathiopirine	ASAT/ALAT normal after 2 months
Han *et al.*^4^	30	M	Fatigue Weakness	ASAT: 400 ALAT: 777 ANA+ ASM–/AM–	No bile duct dilatation	Chronic active hepatitis	Myasthenia	Prednisolone	ASAT/ALAT normal after 1 month
Askawa *et al.*^5^	77	F	Jaundice Ascitis Fatigue	ASAT: 392 ALAT: 280 ANA–/ASM–LKM1–	No bile duct dilatation	Necropsied specimen: auto immune hepatitis	Diabetis Mellitis Myasthenia gravis Hashimoto’s thyroiditis	Vitamin K Plasmapherese	Died from acute hepatitis
Kim *et al.*^6^	35	F	Pruritis Jaundice Fatigue	ASAT: 125 ALAT: 22 ANA+/ASM– LKM1–/AM–	No parenchymal or bile duct abnormalities	Chronic non suppurative destructive cholongitis	No	Ursodeoxy chloric acid	Normal biology for 36 months
Aigner *et al.*^7^	56	F	Loss of appetitis Jaundice	ASAT: 1912 ALAT: 1332 ANA/ASM/	Not mentioned	AIH	Myasthenia gravis after AIH revealing thymoma	Predisone	Normal biology for 48 months
Yapali *et al.*^8^	31	M	Jaundice Fatigue Nausea	ASAT: 370 ALAT: 249 PAL: 152 GT: 208 ANA–/ASM– AM–	No bile duct dilatation	Acute liver hepatitis	Myasthenia gravis Hashimoto’s Thyroiditis Connective tissue disorder	Prednisolone	Normal biology after 6 months

M, male; F, female; ASAT, aspartate aminotransferase; ALAT, alanine aminotransferase; PAL, alkaline phosphatase; GT, gamma glutamyl transferase; AAN, anti-nuclear antibody; ASM, anti smooth muscle antibody; AM, anti mitochondrial antibody; LKM1, anti liver/kidney microsome antibody.

Liver biopsy is always necessary to confirm diagnosis, which relies on the criteria of the International AIH Group^9^. The International AIH Scoring System considers several parameters, such as gender, liver enzymes, antibodies, and concurrent autoimmune disease. Once the patient obtains a score of 15 before treatment or of 17 after treatment, the diagnosis of AIH is definite^9^^,^^10^.

Thymoma is a slow-growing disease that generally leads to liver failure, if not treated, in 50% of cases. The main treatment based on steroids and azathioprine can prevent evolution to cirrhosis. Asakawa *et al.*^5^ reported a case of AIH with rapid liver failure and death in the absence of a specific treatment of thymoma. In this case, the patient did not receive azathioprine because of the high risk of immune deficiency associated with chemotherapy and did not receive steroids because cytolysis was not profound. Cholestasis decreased markedly after chemotherapy alone. In a case reported by Asakawa, the fact that the patient could not be operated on her thymoma may explain the fatal evolution. In this case, chemotherapy allowed complete response on the primary tumor and corrected hepatic disorder.

Our hypothesis is also highlighted by the clinical and molecular data reported by Aigner^7^, who showed the presence of LKM-3 and UGT 1 antibody (UDP glutamyl-transferase involved in AIH) in the patient’s serum, liver, and thymoma tissue. After thymectomy, these antibodies were not found in the serum of the patient. This paper showed the paraneoplastic origin of AIH (which occurred before the diagnosis of thymoma) and the effect of the surgical removal of the primary tumor on correcting this disorder. The active chemotherapy drugs are anthracyclines, cisplatine, and cyclophosphamide. New-generation chemotherapy drugs (pemetrexed, docetaxel, and irinotecan) produced controversial results. If the tumor seems responsive after 3 cycles, it is recommended to consolidate with 3 more cycles. Thymoma is a radiosensitive tumor and residual disease can be monitored with complementary mediastinal radiotherapy^11^. Unlike already published cases, multimodality treatment using chemotherapy and radiotherapy was sufficient to control both the primary tumor and the paraneoplastic hepatic disorder of our patient.

## Conclusion

Thymoma is associated with the high rate of paraneoplastic autoimmune diseases among AIH that should be considered in the etiological diagnosis of hepatic disorders in thymoma. In this case, based on chronological arguments, we speculated that AIH and diabetes mellitus are induced by thymoma. Specific treatments of the primary tumor may treat AIH. Immunosuppressors and steroids should be used only in case of severe evolution or a persistent paraneoplastic disorder.
